# Hepatitis E infection among Ghanaians: a systematic review

**DOI:** 10.1186/s40249-017-0239-9

**Published:** 2017-02-06

**Authors:** Richard Ofori-Asenso, Akosua Adom Agyeman

**Affiliations:** Research Unit, Health Policy Consult, P. O. Box WJ 537, Weija-Accra, Ghana

**Keywords:** Hepatitis E, HEV, Infectious diseases, Viral hepatitis, Systematic review, Ghana

## Abstract

**Background:**

Hepatitis E virus (HEV) infection is considered to be of significant public health importance in many developing countries. In this review, we aim to summarise studies on HEV with the aim of providing a further understanding of the epidemiology of the disease in Ghana.

**Methods:**

A systematic review was conducted by following the recommendations outlined in the PRISMA statement. Studies on HEV infection among Ghanaians were identified by searching for articles (published up until 4th August 2016) in the PubMed, ISI Web of Science, African Journals Online, Google Scholar and the WHO African Index Medicus databases. We also searched the websites of the Ministry of Health and the Ghana Health Service to identify any related non-indexed studies. References of all retrieved studies were screened to identify additional publications.

**Results:**

Ten studies involving a total of 2 894 participants from six regions of Ghana were identified. The proportion of Ghanaians showing positive serological markers for HEV infection was within the range of 5.8–71.55%. In addition, 0.7–45.9% tested positive for IgM antibodies while the proportion of Ghanaians testing positive for IgG antibodies was within the range of 0–45.3%. One study reporting a case fatality rate of 66.7% among pregnant women was identified. No information on HEV genotypes was retrieved.

**Conclusions:**

Although based on a limited number of studies, this review does highlight that there is a high level of HEV infection among Ghanaians. Preventive measures including educational interventions as well as general improvements to sanitary and living conditions are needed to reduce the burden of the disease. Additionally, further research regarding the contribution of the various HEV genotypes is urgently needed to fully understand the burden of this disease in Ghana.

**Electronic supplementary material:**

The online version of this article (doi:10.1186/s40249-017-0239-9) contains supplementary material, which is available to authorized users.

## Multilingual abstracts

Please see Additional file 1 for translations of the abstract into the six official working languages of the United Nations.

## Background

Types of viral hepatitis (A, B, C, D and E) that cause acute and/or chronic infection and liver inflammation are considered to be of significant global public health importance [1]. Hepatitis E (HEV) infection has long been regarded as being endemic in resource-poor regions such as Africa and Southeast Asia, where frequent outbreaks occur due to poor sanitation and hygiene [2, 3]. In more advanced countries, sporadic cases of acute HEV have been traced to individuals with a past history of travel to endemic areas [4]. According to the World Health Organization (WHO), more than 20 million HEV infections occur annually across the world resulting in over 3 million symptomatic cases of hepatitis E [2]. Hepatitis E does not usually develop into a carrier state and most individuals recover fully from acute HEV infections [2, 5]. In a few instances, however, it may progress into fulminant hepatitis, with a case fatality rate of around 1–2% in the general population [6] and as high as 40% in pregnant women [7].

The 2010 Global Burden of Disease Study estimated that there were more than 56 000 HEV-related deaths annually [8]. Although, there is wide acceptance that HEV is of significant public health importance in Ghana, the burden of the disease has not been thoroughly documented [9]. In this paper, we review studies on HEV infection in Ghana to summarise the available evidence regarding incidence and prevalence of the disease. To our knowledge, this is the first systematic review that has attempted to summarise the prevalence and incidence of HEV infection in Ghana. This paper forms part of an ongoing work aimed at documenting the burden of common viral hepatitis in Ghana. Studies on the estimated burdens of other hepatitis types arising from this broad work have been published elsewhere [10, 11].

## Methods

We conducted a systematic review of literature on HEV infection by following the recommendations outlined in the Preferred Reporting Items for Systematic Reviews and Meta-Analyses (PRISMA) statement [12]. Studies reporting HEV infection among Ghanaians were identified by searching for articles (published up to 4th August 2016) in the PubMed, ISI Web of Science, Google Scholar, African Journals Online and the WHO African Index Medicus databases.

The keywords used were ‘Hepatitis E’ OR ‘Hepatitis E virus’ OR ‘HEV’ OR ‘Hepatitis E antibody’* OR ‘non-A and non-B (nAnB)’ AND ‘epidemiology’ OR ‘prevalence’ OR ‘incidence’ AND ‘Ghana’. ‘Non-A’ and non-B (nAnB)’ were included as search terms because HEV has been identified as the causative agent in many epidemic and sporadic cases of enterically transmitted non-A and non-B viral hepatitis in a number of developing countries [13, 14]. We also searched the websites of the Ministry of Health (http://www.moh-ghana.org/) and the Ghana Health Service (http://www.ghanahealthservice.org/) to identify non-indexed studies and policy reports on the subject.

In this review, we included serological studies that reported on the detection of antibodies to HEV-immunoglobulin G (IgG) or immunoglobulin M (IgM) among Ghanaians. The detection of IgM was considered to represent sporadic acute hepatitis cases [5, 15] and was used to estimate the incidence of HEV infection. The detection of IgG was considered to represent remote infection and was used to estimate HEV seroprevalence among the population [15]. Studies assessing case fatality rate were also included.

A 12-point scoring system, adapted from the Downs and Black checklist [16], was used to assess the quality of the individual studies. The scoring system included questions such as whether study’s objective was clearly defined, whether participant characteristics and whether study’s conclusion was sound and based on the results. Descriptive data such as the study population, year of publication, region, setting and the proportion of people who tested positive for IgG or IgM antibodies were summarised. Due to the heterogeneity of the studies, a formal meta-analysis was not conducted.

## Results

**Fig. 1:**
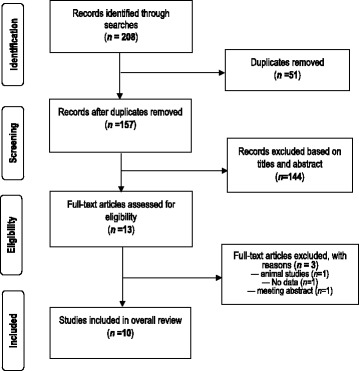
PRISMA flow chart showing the process of selecting studies

Figure 1 summarises the steps used to retrieve the appropriate studies. A total of 208 articles were retrieved. After the titles and abstract were screened and duplicates were removed, 13 studies were selected for full text analysis. Out of these, 10 studies on HEV in Ghana were selected for the review [9, 17–25].

The selected studies (see Table 1) included nine 9 serological studies that reported on the presence of HEV antibodies and one study that focused on case fatality rate. Taken together, the studies involved a total sample population of 2 894 across six regions of Ghana. The regional distribution of studies was as follows: Ashanti (*n* = 3), Greater Accra (*n* = 6) and one study that involved five regions (Ashanti, Brong-Ahafo, Upper East, Upper West and Northern). The majority of the studies (80%, *n* = 8) were conducted solely among urban dwellers. Of the serological studies, one was conducted exclusively among children [17], two studies were conducted among pig handlers [18, 19], two among blood donors [20, 21], one involved adult HIV patients [22], one involved pregnant women [9], one involved persons with viral haemorrhagic fever symptoms [23] and one study involved patients with suspected hepatitis [24]. The ten studies were published between 1999 and 2016, however, the sampling took place between 1993 and 2013. In terms of the quality, 80% (*n* = 8) of the studies were deemed as being of high quality and 20% (*n* = 2) were considered to be of medium quality.

**Table 1:** Studies on hepatitis E in Ghana

Serological Studies reporting markers of HEV infection													
Study No.	Reference	Publication year	Sampling year	Design	Region	Sample size	Setting	Participants	Age of participants	Anti -HEV (%)			Quality grade
										Total Ig	IgG	IgM	
1	Martinson et al. [17]	1999	1993	Cross sectional	Ashanti	803	Rural	Children	6-18	4.4	n.s	n.s	High
2	Adjei et al. [9]	2009	2008	Cross sectional	Greater Accra	157	Urban	Pregnant women	13-42	28.66	10.19	18.47	High
3	Adjei et al. [18]	2009	2008	Cross sectional	Greater Accra	105	Urban	Pig handlers	12–65	38.1	0.0	38.1	High
4	Adjei et al. [19]	2010	2008	Cross sectional	Greater Accra	353	Urban	Pig handlers	15-70	38.48	19.26	15.58	High
5	Tettey [20]	2011	2008	Cross sectional	Greater Accra	471	Urban	Blood donors	18-60 (m) 19–42 (f)	71.55	25.6	45.9	Medium
6	Meldal et al. [21]	2012	n.s	Cross sectional	Ashanti	239	Urban	Blood donors	n.s	10.5	4.6	5.9	High
7	Feldt et al. [22]	2013	2008-10	Cross sectional	Ashanti	402	Urban	Adult HIV patients	40 (±9.6)	46.02	45.3	0.7	High
8	Bonney et al. [23]	2013	2009-13	Hospital-based surveillance study	Ashanti, Brong-Ahafo, Northern, Upper West, Upper East	258	Mixed	Patients with VHF symptoms	23.5^a^	9.7	5.8	3.9	High
9	Ofosu-Appiah et al. [24]	2016	2010-11	Cross-sectional	Greater Accra	103	Urban	Patients with suspected hepatitis	33.0 ± 14.5	5.8	n.s	n.s	Medium
Studies reporting Case Fatality Rate													
	Reference	Publication year	Sampling year	Design	Region	Sample size	Setting	Participants	Age of participants	Case fatality-rate			Quality grade
10	Bonney et al. [25]	2012	2010	Case series	Greater Accra	3	Urban	Pregnant women	31, 31, 17	66.7%			High

*VHF* viral haemorrhagic fever; *n.s* not specified; *m* male; *f* female; *IgG* Immunoglobulin G; *IgM* immunoglobulin M; *HEV RNA* hepatitis E virus RNA

^a^ median

### Serological markers of HEV infection

Among the nine serological studies, the proportion of participants testing positive for any HEV antibody (total Ig) varied from 5.8% to 71.55%. The incidence of HEV infection as determined by the proportion of participants across the seven studies showing positive results for IgM was within the range of 0.7–45.9%. The highest incidence of HEV as determined by IgM seropositivity was reported for a cohort of blood donors (45.9%) [20] and pig handlers (38.1%) [18]. The only study conducted among HIV patients, however, reported a very low incidence rate, with the proportion of participants testing positive for IgM at 0.7% [22]. The seroprevalence of HEV infection (as determined by the proportion of individuals testing positive for IgG) across the seven studies was within the range of 0–45.3%. Seroprevalence of 0% was reported in a cohort of pig handlers [18] and 45.3% in a cohort of HIV-positive adults [22].

### Case fatality rate

Only one study reporting case fatality was identified. It reported a case fatality rate of 66.7% among pregnant women [25]. However, the study involved a small sample size of only three cases.

## Discussion

To our knowledge, this is the first systematic review that has attempted to thoroughly summarise evidence on the incidence and seroprevalence of HEV infection in Ghana. Our results indicate that HEV infection in Ghana may be quite high, with between 5.8% and 71.55% of Ghanaians showing serological markers of past or current infection in the studies under review. The discrepancies in the proportion of Ghanaians testing positive for HEV serological markers across the studies could be related to geographical (regional) differences in the levels of HEV infection across the country, as well as to the varied populations studied.

Almost 60% of the serological studies reported a higher proportion of participants testing positive for IgM than IgG. This by inference suggests that there is a higher HEV incidence compared to prevalence among Ghanaians, which may point to frequent outbreaks than a usual or common situation (usually IgG is higher than IgM). In HIV individuals, while the incidence (based on IgM detection) appears to be very low, the prevalence is very high. This may suggest that HEV infection in HIV-positive Ghanaians persists for longer periods. This longer persistence, which can sometimes lead to chronic states, is also increasingly being reported globally [26, 27]. This trend is explained by the limited immune system capacity of HIV patients to resolve acute infections and HEV may be regarded as just another form of opportunistic infection [27].

Only one study [9] was conducted among pregnant women, which reported that 28.7% showed serological markers of HEV infection. The highest proportion comprised women in the third trimester – an observation that is consistent with globally reported trends [28]. HEV infection in pregnant women may pose additional health risks due to the possible vertical transmission of the disease.

The two studies that focused on blood donors reported that 10.5% and 71.55% tested positive for serological markers of HEV infection. While the clinical relevance of transfusion-transmitted HEV remains unclear [29], studies have demonstrated the possibility of HEV transmission through blood transfusions in endemic areas, following retrospective evaluations of transfusion recipients [30]. As such, the high levels of HEV infection among blood donors in Ghana could pose serious concerns regarding the safety of blood supply [31]. Although, Ghana has a mandatory policy that requires the screening of all donated blood, this mainly covers HIV 1 and 2, hepatitis B, hepatitis C and syphilis [32]. Therefore, attention must also be paid to HEV, as the risk of spreading the virus through blood transfusion could be high, as this review suggests.

The studies conducted among pig handlers reported that over 38% showed serological markers of HEV infection. Adjei *et al.* [19] found that among pig handlers, the risk of HEV infection correlated with the level of contact with animals or their waste, and the absence of piped water on the farms. Although the studies on pig handlers in Ghana did not report on a particular genotype, studies elsewhere have suggested possible zoonotic transmission of HEV, especially of genotypes 3 and 4 [33, 34].

The study by Bonney *et al*. [25], although based on a small sample size (*n* = 3), reported an HEV case fatality rate of 66.7% among pregnant women in Ghana. This rate is high when compared with the case fatality rate reported in Sudan (31.1%) [35] and the Central African Republic (14.3%) [36], although perhaps it is better to do these comparisons when evidence of the HEV genotype becomes available.

The seemingly high levels of HEV infection among Ghanaians may be attributed to the low level of knowledge and awareness about the transmission pathways of common viral hepatitis in the country [37, 38]. Additionally, many communities in Ghana are densely populated and sanitation conditions remain precarious, with only around 13% of Ghanaians estimated to have access to improved sanitation [39]. All this may facilitate the outbreak of HEV, particularly of genotypes 1 and 2 [2, 3, 9]. There is evidence of high faecal-oral transmission of HEV in African countries [40], which suggests that public health measures may need to address issues such inadequate access to safe water and poor sanitation in an effort to control HEV outbreaks.

Currently, there is no specific treatment available for HEV infection. However, a recombinant vaccine to prevent the disease has been developed and recently approved in China, but is not yet available in any other country [41]. Early studies on this vaccine have demonstrated 95% efficacy in preventing HEV infection and clinical disease [42, 43]. Once this vaccine becomes widely available, it may be particularly useful for high-risk groups such as HIV patients, in whom HEV infection remains high.

This study had some limitations. The main one is the relatively small number of studies identified. This highlights that research and an understanding of the HEV infection burden in Ghana is less developed compared to research on and understanding of other viral infections. The majority of the studies (80%, *n* = 8) involved samples from only two regions of Ghana. Subsequently, no samples from four regions (Central, Volta, Eastern and Western) were included in the studies reviewed. Additionally, there are four major genotypes of hepatitis with different modes of transmission [44] and which may require different approaches for control. The studies reviewed, however, did not report on these genotypes. Furthermore, there are challenges in diagnosing HEV infections and while there are many diagnostic assays, not all have been rigorously tested and may at times present incongruous results [45]. For instance, currently the US Food and Drug Administration has not approved any serologic test for HEV diagnosis [46].

## Conclusions

Although this review is based on a limited number of studies, it does highlight a high level of HEV infection among Ghanaians. Preventive measures including educational interventions, screening of all donated blood and high-risk groups (e.g., HIV patients), as well as general improvement in sanitary and living conditions are needed to reduce the burden of the disease. Additionally, further research regarding the contribution of the various HEV genotypes is urgently needed to fully understand the burden of the disease in Ghana.

## Additional file

Additional file 1: Multilingual abstracts in the six official working languages of the United Nations. (PDF 712 kb)

## Abbreviations

HEV: Hepatitis E virus
IgG: Immunoglobulin G
IgM: Immunoglobulin M
PRISMA: Preferred Reporting Items for Systematic Reviews and Meta-Analyses
WHO: World Health Organization.
