# Supplementary material for: Hepatitis E infection among Ghanaians: a systematic review
Source: Infect Dis Poverty. 2017 Feb 6;6:29. doi: 10.1186/s40249-017-0239-9 (PMC5292811; doi:10.1186/s40249-017-0239-9)
Supplement: Additional file 1: — Multilingual abstracts in the six official working languages of the United Nations. (PDF 712 kb) [file 40249_2017_239_MOESM1_ESM.pdf]

Translation of the abstract into the six official working languages of the United Nations

عدوى التهاب الكبد ه بين الغانيين: مراجعة منهجية

ريتشارد أوفوري-أسينسو، أكوسوا أدوم أجييمان

ملخص
خلفية: تعتبر عدوى فيروس التهاب الكبد الوبائي ه ذات أهمية كبيرة على الصحة العامة في العديد من البلدان النامية. في هذا الاستعراض، نهدف إلى تلخيص دراسات حول فيروس التهاب الكبد الوبائي ه بهدف توفير مزيد من الفهم لوبائيات المرض في غانا.
الطرق: أجريت مراجعة منهجية باتباع التوصيات الواردة في بيان PRISMA. وحددت الدراسات على عدوى فيروس التهاب الكبد الوبائي ه بين الغانيين من خلال البحث عن المواد (المنشورة حتى 4 أغسطس 2016) قواعد بيانات مجلات PubMed وISI Web of Science وAfrican Journals Online و Google Scholarو WHO African Index Medicus. وبحثنا أيضا في المواقع التابعة لوزارة الصحة والخدمات الصحية غانا لتحديد أي دراسات غير المفهرسة ذات الصلة. تم فحص المراجع لجميع الدراسات التي تم الوصول إليها لتحديد منشورات إضافية.
النتائج: قد تم تحديد عشرة (10) دراسات شملت ما مجموعه 2894 مشاركا من ستة (6) مناطق في غانا. وكانت نسبة الغانيين الذين تظهر عليهم علامات مصلية إيجابية للإصابة بالتهاب في حدود 5.8-71.55٪. وبالإضافة إلى ذلك، كان نسبة 0.7-45.9٪ إيجابية للأجسام المضادة للجلوبولين المناعي في حين كانت نسبة الغانيين ممن لديهم مضادات الجلوبولين في نطاق 0-45.3٪. وقد حددت إحدى الدراسات معدل إماتة الحالات ليكون 66.7٪ بين النساء الحوامل. لم يتم الحصول على أي معلومات عن مورثات فيروس التهاب الكبد الوبائي ه.
الاستنتاجات: على الرغم من اعتماد الاستعراض على عدد محدود من الدراسات، فإنه لا يبرز أن هناك مستوى عال من عدوى فيروس التهاب الكبد الوبائي ه بين الغانيين. وهناك حاجة إلى اتخاذ تدابير وقائية بما في ذلك التدخلات التعليمية وكذلك تحسينات عامة على الظروف الصحية والمعيشية للحد من عبء المرض. بالإضافة إلى ذلك، هناك حاجة ملحة لإجراء المزيد من البحوث فيما يتعلق بمساهمة مختلف الأنماط الوراثية لفيروس التهاب الكبد الوبائي ه للتوصل إلى فهم كامل لعبء هذا المرض في غانا.

Translated from English version into Arabic by Mahmoud Sami, through

TRANSLATORS
WITHOUT BORDERS

加纳人群戊型肝炎病毒感染情况：系统综述

Richard Ofori-Asenso, Akosua Adom Agyeman

摘要
引言：在许多发展中国家，戊型肝炎病毒（HEV）感染对公共卫生具有重要意义。本综述旨在总结加纳 HEV 相关研究以进一步提供其流行病学资料。
方法：本文按照 PRISMA 声明的建议进行了系统综述。我们在 PubMed、ISI Web of Science、非洲期刊在线、谷歌学术和 WHO 非洲指数药物数据库中搜索并挑选加纳人群 HEV 感染研究论文（发表日期截至 2016 年 8 月 4 日）。且在加纳卫生部和卫生服务网站上搜索资料以纳入任何相关非索引性研究；筛选所有检索研究的参考文献以确定其他出版物。
结果：确定了 10 项涉及加纳 6 个地区，共计 2 894 名参与者的研究。结果显示，加纳人群血清学阳性比例为 5.80%-71.55%。此外，0.7%-45.9%的加纳人 IgM 抗体阳性，而 IgG 抗体阳性比例在 0-45.3%之间。确定了一项报道孕妇病死率为 66.7%的研究报告。没有检索到 HEV 基因型相关信息。
结论：尽管研究数量有限，本综述突出显示了加纳人群中存在高水平的 HEV 感染。减轻当地的疾病负担需要采取预防措施，包括教育干预、大致改善卫生和生活条件。此外，急需进一步研究各种 HEV 基因型作用以充分了解加纳 HEV 疾病负担。

Translated from English version into Chinese by Jin Chen, edited by Pin Yang

CHINESE CENTER FOR DISEASE CONTROL AND PREVENTION
NATIONAL INSTITUTE OF PARASITIC DISEASES

Infection par l'hépatite E parmi les Ghanéens: une revue systématique

Richard Ofori-Asenso, Akosua Adom Agyeman

RÉSUMÉ
Contexte: le virus de l'hépatite E (VHE) est considéré comme revêtant une importance significative pour la santé publique dans de nombreux pays en voie de développement. La présente revue a pour objectif de synthétiser les études menées sur le VHE afin de permettre une compréhension approfondie de l'épidémiologie de la maladie au Ghana.
Méthodes: une revue systématique a été menée conformément aux recommandations stipulées dans la déclaration du PRISMA. Des études relatives à l'infection par le VHE parmi les Ghanéens ont été identifiées dans le cadre de la recherche d'articles (publiés jusqu'au 4 août 2016) dans les bases de données PubMed, ISI Web of Science, African Journals Online, Google Scholar et l'Index Medicus Africain de l'OMS. Nous avons aussi effectué des recherches sur les sites web du Ministère de la Santé et des Services de santé du Ghana afin d'identifier toute étude liée et non référencée. Les références de toutes les études examinées ont fait l'objet d'un passage en revue afin d'identifier des publications supplémentaires.
Résultats: dix (10) études impliquant au total 2894 participants provenant de six (6) régions du Ghana ont été identifiées. La proportion de Ghanéens présentant des marqueurs sérologiques positifs indiquant une infection par le VHE s'étendait de 5,8 à 71,55 %. De plus, des anticorps IgM ont été détectés chez 0,7 à 45,9 % des Ghanéens, tandis que la proportion de Ghanéens ayant obtenu un résultat positif au test de dépistage des anticorps IgG s'étendait, elle, de 0 à 45,3 %. Une étude faisant état d'un taux de létalité de 66,7 % chez les femmes enceintes a été identifiée. Aucune information sur les génotypes du VHE n'a été recueillie.
Conclusions: bien qu'elle ne repose que sur un nombre restreint d'études, cette revue souligne bien l'existence d'un taux élevé d'infection par le VHE parmi les Ghanéens. Des mesures de prévention ainsi que des interventions de sensibilisation et des améliorations générales des conditions de salubrité et de vie sont requises pour réduire le fardeau de la maladie. De plus, des recherches approfondies portant sur la contribution des divers génotypes du VHE sont impérativement nécessaires afin d'obtenir une compréhension approfondie du fardeau de cette maladie au Ghana.

Translated from English version into French by eric ragu, through

TRANSLATORS
WITHOUT BORDERS

Вирус гепатита Е в Гане– систематический обзор

Richard Ofori-Asenso, Akosua Adom Agyeman

РЕФЕРАТ
Введение: Врачи многих развивающихся стран рассматривают вирус гепатита Е как одну из наиболее серьезных проблем в плане охраны здоровья. В настоящем обзоре авторы попытались обобщить исследования по вопросу заболеваемости гепатитом Е, с тем чтобы обеспечить более полное понимание сложившейся в Гане эпидемиологической картины.
Методы: Систематический обзор проводился с учетом рекомендаций относительно предпочтительных параметров отчетности для систематических обзоров и метаанализа (PRISMA Statement). Для выявления массива исследований по вопросам распространения гепатита Е в Гане поиск публикаций (дата публикации – до 4 августа 2016 года) был проведен по следующим источникам: PubMed, ISI Web of Science, African Journals Online, Google Scholar и базы данных ВОЗ African Index Medicus. Кроме того, чтобы выявить непроиндексированные публикации, авторы провели поиск по веб-сайтам Министерства здравоохранения и Службы охраны здоровья Ганы. Ссылки, приведенные во всех выявленных публикациях, были проанализированы на предмет выявления дополнительных публикаций.
Результаты: Было выявлено 10 (десять) исследований, в которых приняли участие 2894 человека из 6 (шести) областей Ганы. Доля жителей Ганы, давших положительную реакцию на серологические маркеры наличия вируса гепатита Е, составила от 5,8 до 71,55 процента. Кроме того, в 0,7–45,9% случаев были выявлены антитела IgM, а доля носителей антител IgG среди участвовавших в исследованиях жителей Ганы составила от 0 до 45,3 процента. Согласно одному из исследований, среди беременных женщин в 66,7% случаев наблюдался смертельный исход. Информации о распространении отдельных генотипов вируса гепатита Е выявлено не было.
Выводы: Несмотря на ограниченное число выявленных исследований, по результатам обзора было установлено, что число инфицированных вирусом гепатита Е в Гане высоко. Чтобы одолеть бремя болезни, необходимы профилактические меры, в том числе мероприятия образовательного характера, общее повышение уровня санитарии

и гигиены и улучшение условий жизни. Кроме того, чтобы достичь полного понимания сложившейся в Гане ситуации, необходимо незамедлительно провести исследования, которые позволят определить распространение в стране отдельных генотипов вируса гепатита Е.

Translated from English version into Russian by Alexander Poddubnyy, through

TRANSLATORS
WITHOUT BORDERS

La infección por hepatitis E entre la población de Ghana: Una revisión sistemática

Richard Ofori-Asenso, Akosua Adom Agyeman

RESUMEN
Antecedentes: La infección por el virus de la hepatitis E (VHE) se considera un asunto de salud pública muy importante en muchos países en desarrollo. En la presente revisión, nuestro objetivo es resumir los estudios sobre el VHE con el fin de proporcionar una mayor comprensión de la epidemiología de la enfermedad en Ghana.
Métodos: La revisión sistemática se realizó siguiendo las recomendaciones esbozadas en la declaración PRISMA. Los estudios sobre la infección por VHE entre la población de Ghana se identificaron mediante la búsqueda de artículos (publicados hasta al 4 de agosto de 2016) en las bases de datos de *PubMed*, de la *Web of Science* del ISI (*Institute for Scientific Information*), del *African Journals Online*, de *Google Scholar* y del Index Medicus de la Región de África de la OMS. También se realizaron búsquedas en las páginas web del Ministerio de Salud y del Servicio de Salud de Ghana con el fin de identificar cualquier estudio no indexado relacionado. Se examinaron las referencias de todos los estudios obtenidos para identificar publicaciones adicionales.
Resultados: Se identificaron diez (10) estudios que incluían un total de 2.894 participantes de seis (6) regiones de Ghana. La proporción de ghaneses que presentaba marcadores serológicos positivos de la infección por VHE se hallaba dentro de la gama del 5,8 % al 71,55 %. Adicionalmente, la proporción de ghaneses que dieron positivo en anticuerpos de IgM se sitúa entre el 0,7 % y el 45,9 %, mientras que la proporción que dieron positivo en anticuerpos IgG estaba dentro del rango de 0 a 45,3 %. Uno de los estudios informaba de una tasa de mortandad del 66,7 % entre mujeres embarazadas. No se recuperó ningún tipo de información sobre genotipos VHE.
Conclusiones: A pesar de estar basada en un número limitado de estudios, la presente revisión destaca la existencia de un alto nivel de infección por VHE entre la población de Ghana. Es necesario tomar medidas preventivas que incluyan actuaciones educativas, así como mejoras generales en las condiciones sanitarias y de vida para reducir la carga que supone esta enfermedad. Además, urge llevar a cabo una investigación más amplia sobre la contribución de los diferentes genotipos del VHE para comprender plenamente la carga que representa esta enfermedad en Ghana.

Translated from English version into Spanish by Pilar Daza, through

TRANSLATORS
WITHOUT BORDERS
